# Crescent-Like Lesions as an Early Signature of Nephropathy in a Rat Model of Prediabetes Induced by a Hypercaloric Diet

**DOI:** 10.3390/nu12040881

**Published:** 2020-03-25

**Authors:** Sara Nunes, André Alves, Inês Preguiça, Adelaide Barbosa, Pedro Vieira, Fernando Mendes, Diana Martins, Sofia D. Viana, Flávio Reis

**Affiliations:** 1 Institute of Pharmacology & Experimental Therapeutics, & Coimbra Institute for Clinical and Biomedical Research (iCBR), Faculty of Medicine, University of Coimbra, 3000-548 Coimbra, Portugal; 2 Center for Innovative Biomedicine and Biotechnology (CIBB), University of Coimbra, 3004-504 Coimbra, Portugal; 3 Polytechnic Institute of Coimbra, ESTESC-Coimbra Health School, Pharmacy/Biomedical Laboratory Sciences, 3046-854 Coimbra, Portugal; 4 Biophysics Institute & Coimbra Institute for Clinical and Biomedical Research (iCBR) area of Environment Genetics and Oncobiology (CIMAGO), Faculty of Medicine, University of Coimbra, 3000-548 Coimbra, Portugal; 5 i3S—Institute for Research and Innovation in Health, University of Porto, 4200-135 Porto, Portugal

**Keywords:** prediabetes, nephropathy, rat model, diet-induced, renal lipidosis, glomerular crescent-like lesions

## Abstract

Diabetic nephropathy (DN) is a major microvascular complication of diabetes. Obesity and hyperlipidemia, fueled by unhealthy food habits, are risk factors to glomerular filtration rate (GFR) decline and DN progression. Several studies recommend that diabetic patients should be screened early (in prediabetes) for kidney disease, in order to prevent advanced stages, for whom the current interventions are clearly inefficient. This ambition greatly depends on the existence of accurate early biomarkers and novel molecular targets, which only may arise with a more thorough knowledge of disease pathophysiology. We used a rat model of prediabetes induced by 23 weeks of high-sugar/high-fat (HSuHF) diet to characterize the phenotype of early renal dysfunction and injury. When compared with the control animals, HSuHF-treated rats displayed a metabolic phenotype compatible with obese prediabetes, displaying impaired glucose tolerance and insulin sensitivity, along with hypertriglyceridemia, and lipid peroxidation. Despite unchanged creatinine levels, the prediabetic animals presented glomerular crescent-like lesions, accompanied by increased kidney Oil-Red-O staining, triglycerides content and mRNA expression of IL-6 and iNOS. This model of HSuHF-induced prediabetes can be a useful tool to study early features of DN, namely crescent-like lesions, an early signature that deserves in-depth elucidation.

## 1. Introduction

According to estimates of the International Diabetes Federation (IDF), the prevalence of type 2 diabetes mellitus (T2DM) will continue to grow for decades to come in many world regions, as well as its serious complications, including diabetic nephropathy (DN), which affects around one-third of patients with diabetes, imposing major socio-economic costs [1,2].

Although T2DM evolves slowly and silently until diagnosis, it progressively affects the microvasculature, including the kidney tissue [3,4]. Glomerular hypertrophy, a modest expansion of mesangial matrix, and thickening of glomerular capillary walls are the main features of the initial stages of DN, then evolving to a phenotype of glomerulosclerosis, fueled by thickening of glomerular basement membrane (GBM), mesangial cell expansion and loss of podocyte, along with tubulointerstitial fibrosis, reduction of glomerular filtration rate (GFR), and albuminuria [5]. Unfortunately, despite the great scientific advances, the current panoply of glucose-lowering drugs has been clearly insufficient to hinder diabetic microvascular complications, including DN [6,7]. Thus, early identification (and intervention) of diabetic patients with renal impairment is urgently needed.

Prediabetes, also termed intermediate hyperglycemia, has been recognized as a risk factor for the development of both T2DM and DN [8,9,10]. It has been strongly suggested that people with prediabetes should be early screened for kidney disease [11]. However, this mission is certainly very challenging since the classical markers of kidney function, namely serum creatinine and GFR, are influenced by several factors that bias the interpretation, as well as because they might remain unchanged until a high proportion (around 50%) of nephrons become nonfunctional [12,13]. This is a very important concern that has sparked much research in recent years, in order to better characterize the phenotype of initial DN and to identify not only early biomarkers but also putative targets for timely therapeutic intervention, which is a clear unmet clinical need [14]. For this purpose, animal models are a privileged tool with great utility.

Great progress was made during the last two decades to develop better animal models of DN, namely as a result of the major advances in genetic manipulation. Monogenic or polygenic mice models can recapitulate important features of the human pathology, namely when combined with other drivers of disease progression, including the use of (i) streptozotocin (or other toxins) to accelerate diabetes due to promotion of beta-cell damage; (ii) hypercaloric diets (sugar- and/or fat-based); (iii) nephrectomy strategies to reduce kidney mass and aggravate the renal decline and development of lesions (as previously reviewed [15,16,17]). However, no single model is able to present all the functional and structural changes of the human DN [18] and to accomplish the 3 criteria defined in 2003 by the “Animal Models of Diabetic Complications Consortium (AMDCC)” for DN models [19]. Despite the unequivocal utility of genetic models, they present relevant limitations, including the difficulty to obtain, feed and breed, the prolonged modeling cycle, together with the complete loss of gene expression that is rarely observed in human disease [20,21]. Therefore, animal models induced by dietary modifications have become popular, both in rat and mouse [22,23,24,25,26], because they are based on a major risk factor for the human disease: unhealthy food habits. Hypercaloric dietary regimens are chief promotors of obesity and hyperlipidemia, which exacerbate the risk of GFR decline and DN progression [27,28,29,30]. In addition, evidence supports the existence of abnormal deposition of lipids in the kidney (renal lipidosis) in patients with early stage DN [31], which might promote an inflammatory response that seems to play a key role in the onset and progression of DN [32]. However, the impact of this deposition on markers of renal function and on the histomorphological phenotype of early renal disease remain to be clearly elucidated, namely in the stage of prediabetes. In this study, we used a rat model with prediabetes induced by high sugar and high fat (HSuHF) diet to characterize the phenotype of early renal dysfunction.

## 2. Methods

### 2.1. Animals and Experimental Design

Male Wistar rats (eight-week-old; Charles River Laboratories, Barcelona, Spain) were housed two per cage in the animal facility of Coimbra Institute for Clinical and Biomedical Research (iCBR), Faculty of Medicine, University of Coimbra, and were maintained under controlled temperature (22 ± 1 °C), relative humidity (50–60%) and a 12-h light/dark cycle conditions. All procedures involving animals were performed according to the National and European Communities Council Directives of Animal Care. The project received approval (9/2018) by the local (iCBR) Animal Welfare Body (ORBEA).

After an adaptation period of 1 week, rats were randomly divided into two groups (*n* = 8 each, 2 animals per cage) and submitted to a 23-weeks protocol: Control group—received standard rat chow (total 3.15 kcal^−1^—8.6% of energy as fat, 67.9% as carbohydrates, 23.5% as protein; 4RF21 Mucedola, Milan, Italy) and tap water; HSuHF group—received 35% sucrose (Hsu-84100; Sigma-Aldrich, Saint Louis, Missouri, USA) in the drinking water plus standard chow until week 9, further supplemented by High Fat diet (HF, total 5.10 kcal g^−1^—61.6% of energy as fat, 20.3% as carbohydrates and 18.1% as protein; 58Y1, TestDiet) until week 23.

Animals consumed food and water *ad libitum* (except during fasting periods). Body weight (BW) measurements were obtained weekly using an analytical balance (CQT 2000 Core^®^ Portable Compact Balance, Adam Equipment, Oxford, CT, USA) and food and liquid consumption were also monitored weekly.

### 2.2. Blood and Tissues Collection

The day before finishing the experiment (at week 23), rats were submitted to an intraperitoneal anesthesia with a 2 mg/kg BW of a 50 mg/kg pentobarbital (Sigma-Aldrich, Saint Louis, Missouri, USA) solution and a blood sample was instantly collected by venipuncture from the jugular vein into syringes (for serum samples collection). Thereafter, the rats were sacrificed by cervical dislocation and kidneys were immediately collected, weighed and divided, and stored according to further analysis: prefixed in a 10% neutral buffered formalin solution for histopathological analysis or cryopreserved in liquid nitrogen for triglycerides content and lipid peroxidation assays, protein and gene expression analysis.

### 2.3. Metabolic Characterization

#### 2.3.1. Glycemic Profile

Fasting and fed glycemia: Blood glucose levels at fasting (assessed after 6-h of fasting, performed between 8:00 and 14:00) and under fed conditions were measured in the sacrifice day from a drop of blood collected by venipuncture in the jugular vein with the aid of the Accu-Chek^®^ Aviva glucometer (Roche, Mannheim, Germany).

Hemoglobin A1C (HbA1c) was measured on the day of sacrifice through a drop of blood placed on an automated analyzer (Siemens, DCA Vantage Analyzer TM, Tarrytown, NY, USA).

Glucose tolerance test (GTT): In the first day of the last treatment week (23), rats were administered intraperitoneally with a glucose bolus of 2 g/kg BW following a 6-h fasting period. Blood glucose levels were quantified through the tail vein before the injection and 15, 30, 45 and 60 min after, using the portable device Accu-Chek^®^ Aviva glucometer (Roche, Mannheim, Germany). The area under the curve (AUC) for the GTT was calculated by using the trapezoidal method, as previously described [23].

#### 2.3.2. Insulinemic Profile

Fasting and fed insulin: Insulin levels were measured in serum samples (in fed and 6-h fasting conditions, performed between 8:00 and 14:00) by using a rat insulin ELISA (Enzyme-Linked Immuno-Sorbent Assay) kit from Mercodia (Uppsala, Sweden).

Insulin tolerance test (ITT): In the last day of the last treatment week (23), rats were administered intraperitoneally with insulin (0.75 U/kg BW, Actrapid Novo Nordisk) following a 6-h fasting period. Blood glucose levels were obtained through a drop of blood from the tail vein before the bolus and 30, 60 and 120 min after, using the portable device Accu-Chek^®^ Aviva glucometer (Roche, Mannheim, Germany). The area under the curve (AUC) for the ITT was calculated by using the trapezoidal method, as previously described [24].

#### 2.3.3. Lipidic Profile

At the end of the in vivo protocol, blood was collected into syringes without anticoagulant and centrifuged (35,000 rpm, 15 min at 4 °C). Total cholesterol and TG levels were assessed in serum using an automated analyzer (Hitachi 717, Roche Diagnostics, Mannheim, Germany) that makes use of Cholesterol RTU reagent and kit TG PAP 1000 (bioMérieux, Lyon, France) for the measurement of cholesterol and TGs, respectively, by colorimetric methods.

### 2.4. Serum Markers of Redox Status and Inflammation

Serum redox status was assessed by the thiobarbituric acid reactive species (TBARs) assay, measuring lipid peroxidation via malondialdehyde (MDA) content, and by the total antioxidant status (TAS) quantification, through ferric reducing antioxidant potential (FRAP) assay, as previously described [33]. High sensitivity C-reactive protein (hs-CRP) levels in serum were measured by using a rat-specific Elisa kit (MBS764381, MyBiosource, San Diego, CA, USA).

### 2.5. Renal Function Data

#### 2.5.1. Serum Biochemical Parameters

The following biochemical parameters were evaluated in serum by validated automated methods and equipment (Hitachi 717, Roche Diagnostics, Mannheim, Germany): creatinine, uric acid, and blood urea nitrogen (BUN).

#### 2.5.2. 24-h Urine Parameters

During week 23, animals were housed in metabolic cages for 24 h, receiving water and food *ad libitum.* Urinary creatinine, BUN and uric acid concentrations were evaluated in 24-h urine (Cobas Integra 400 plus, Roche Diagnostics, Mannheim, Germany), and urine volumes were measured to calculate clearances, according to what was previously described [34].

### 2.6. Renal Histomorphology

#### 2.6.1. Hematoxylin and Eosin (H&E) Staining

Renal tissue samples were formalin-fixed and embedded in paraffin wax. Cryosections (5 µm) from each block were reviewed. Briefly, tissue sections were deparaffinized in xylene and hydrated to a decrescent series of ethanol until distilled water. Thereafter, the tissue sections were immersed in hematoxylin stain Solution, Gill 1 (Sigma Aldrich, Saint Louis, MO, USA) for 2 min and washed in tap water. Then, they were counterstained with 0.5% aqueous eosin (Sigma Aldrich; MO, USA) for 30 s and after that dehydrated, cleared, and mounted. All samples from both groups (*n* = 8 for each) were examined by light microscopy using a Zeiss microscope Mod. Axioplan 2 (Göttingen, Germany). The degree of injury visible by light microscopy was scored in the total tissue on the slide, focusing glomerular (100 glomeruli per experimental condition), tubulointerstitial and vascular lesions.

#### 2.6.2. Periodic Acid of Shiff (PAS) and PAS-Diastase Staining

For PAS staining, tissue sections were deparaffinized in xylene and hydrated to a decrescent series of ethanol until distilled water. Then, they were treated with periodic acid (Sigma Aldrich, Saint Louis, MO, USA) for 5 min and rinsed in distilled water. After that they were stained with Schiff’s reagent (Sigma Aldrich, Saint Louis, MO, USA) for 10 min and washed in tap water for 3 min. Nuclei were counterstained with hematoxylin stain Solution, Gill 1 (Sigma Aldrich, Saint Louis, MO, USA) for 1 min and rinsed in tap water. Tissue sections are then dehydrated cleared and mounted.

For Pas-Diastase, tissue sections were deparaffinized in xylene and hydrated to a decrescent series of ethanol until distilled water. Then, they were treated with 0.5% amylase solution (Sigma Aldrich, Saint Louis, MO, USA) for 15 min and washed in running tap water followed by periodic acid incubation (Sigma Aldrich, Saint Louis, MO, USA) for 5 min and rinsed in distilled water. After that they were stained with Schiff’s reagent (Sigma Aldrich, Saint Louis, MO, USA) for 10 min and washed in tap water for 3 min. Nuclei were counterstained with hematoxylin stain Solution Gill 1 (Sigma Aldrich, Saint Louis, MO, USA) for 1 min and rinsed in tap water. Tissue sections were then dehydrated cleared and mounted. All samples were examined by light microscopy using a Zeiss microscope Mod. Axioplan 2 (Göttingen, Germany).

#### 2.6.3. Gomori’s Green Trichrome Staining

Tissue sections were deparaffinized in xylene and hydrated to a decrescent series of ethanol until distilled water. Then the slides were treated with Weigert’s iron hematoxylin (Sigma Aldrich, Saint Louis, MO, USA) stain from 7 min. Slides were placed into Chromotrope 2R (Sigma Aldrich, Saint Louis, MO, USA) at 0.2% with Light green at 0.8% solution for 5 min. Then, a quickly rinse off the stain with distilled water was performed and then differentiated with acetic acid at 0.5%. Tissue sections were then dehydrated, cleared, and mounted. The cytoplasm of the cells was stained in red, fibrin in pink whereas collagen was observed in green, in contrast with blue or black nuclei. All samples were examined by light microscopy using a Zeiss microscope Mod. Axioplan 2 (Göttingen, Germany)

#### 2.6.4. Collagen IV Immunohistochemistry

Renal sections (7 µm in thickness) were cut in a cryostat (Leica CM3050S, Nussloch, Germany), mounted directly onto SuperFrost Plus microscope slides (Menzel-Glaser, Thermo Fisher Scientific, Braunschweig, Germany) and stored at −80 °C until use. Renal sections were air dried for at least 30 min at room temperature (RT) and then fixed in cold acetone:methanol (1:1) for 10 min. After washing with phosphate-buffered saline (PBS: 137 mM NaCl, 27 mM KCl, 81 mM Na2HPO4, 15 mM KH2PO4, pH 7.3, 3 × 5 min), sections were permeabilized for 15 min with 0.5% Triton X-100 in PBS and blocked with 3% BSA in PBS for 40 min. Sections were incubated overnight at 4 °C with primary antibody: anti- collagen IV (1:250, ab19808-Abcam), diluted in PBS with 0.3% BSA. After rinsing with PBS, slices were incubated with secondary fluorescent antibody Alexa Fluor 488-conjugated goat anti-Rabbit (1:200; AB029488—SICGEN) and with 4′,6-diamidino-2-phenylindole (DAPI) for 1 h at RT in a humidified chamber in the dark. Samples were washed with PBS (3 × 5 min), mounted using the glycergel mouting medium (Dako North America, Inc, Carpinteria, CA, USA) and sealed with nail polish. The samples were stored at 4 °C until acquisition of images in the confocal microscope (LSM 710, Carl Zeiss, Göttingen, Germany). For a negative experiment, the primary antibody was omitted.

#### 2.6.5. Ki-67 Immunostaining

Tissue sections were deparaffinized in xylene and hydrated to a decrescent series of ethanol until distilled water. Antigen retrieval was performed using high pH target retrieval solution (Dako, Glostrup, Denmark) in a PTLink module (Dako) for 20 min at 96 °C. Slides were then treated with an endogenous-peroxidase blocking solution (S2023; Dako) followed by a protein blocking solution (X0909; Dako) and the primary antibody at 1:1000: anti-Ki-67 Ab, MIB-5, (Dako M7248). The reaction was detected using the HRP labelled polymer (DakoCytomation) and revealed with diaminobenzidine (DAB) chromogen DakoCytomation).

### 2.7. Renal Lipid Deposition

#### 2.7.1. Oil Red O Staining

Tissue sections of fresh frozen tissue were cut to 5 µm thickness, mounted on slides and allowed to dry for 30 min. The cryosections were placed in absolute propylene glycol for 2 min and transferred to 0.5% red oil in absolute propylene glycol solution for 10 min. The sections were differentiated in 85% propylene glycol solution for 2 min, washed in distilled water and counterstained in Hematoxylin Stain Solution Gill 1 (Sigma Aldrich, Saint Louis, MO, USA) for 30 s. They were rinsed under running water for 3 min and mounted with CC / Mount aqueous mounting medium (Sigma Aldrich, Saint Louis, MO, USA). The lipids were stained with bright red color and nuclei with a blue color. All samples were examined by light microscopy using a Zeiss microscope Mod. Axioplan 2.

#### 2.7.2. Kidney Triglycerides Levels

Triglycerides contents on kidney samples were measured by an enzymatic colorimetric assay using a commercial kit (Ref.1155010, Triglycerides MR, Cromatest ^®^, Linear Chemicals, Barcelona, Spain). Briefly, 50 mg of frozen tissue were homogenized in 1 mL of isopropanol. The homogenate was sonicated and then centrifuged at 3000 rpm for 5 min at 4 °C, and the supernatant was analysed following the manufacter’s instructions.

### 2.8. Kidney Gene and Protein Expression

#### 2.8.1. qRT-PCR Gene Expression Analysis

50 mg from frozen renal tissue (stored in RNA later™ solution, ThermoFisher) were processed with a Trizol protocol (93289, Sigma).and stored overnight at −80 °C. Total amount of RNA extracted, RNA integrity (RIN, RNAIntegrity Number) and purity (A260/A280) were measured by Nano Chip^®^ kit in Agilent 2100 Bioanalyzer (2100 expert software, Agilent Technologies, Walbronn, Germany) and ND-1000^®^ spectrophotometer (NanoDrop Tecnhologies, Wilmington, DE, USA), respectively. cDNA was synthesized using a Xpert cDNA Synthesis Mastermix (GK81.0100, Lot. 7E2709A, GRISP, Porto, Portugal) according to the manufacter’s instructions.

For each PCR reaction, 20 µL volume was used containing 2 µL of cDNA, 10 µL of Sybr Green (iTaq Universal SYBR Green Supermix 1725124, Bio-Rad, Hercules, CA, USA), 0.4 µL of each primer (listed in Table 1) and 7.6 µL of autoclaved DEPC water. Gene expression was normalized with GeNorm algorithm, where gene stability was attained with glyceraldehyde 3-phosphate dehydrogenase (GAPDH) and hypoxanthine-guanine phosphoribosyltransferase (HPRT). The relative expression ratio of each of the target gene was computed on the basis of ∆∆Ct (2^−∆∆Cp^) values.

#### 2.8.2. Protein Content Analysis by Western Blotting

Kidneys were weighed (150–200 mg) and homogenized in ice-cold Radio Immuno Precipitation Assay (RIPA) buffer. Thereafter, samples were centrifuged at 13,200 rpm for 15 min at 4 °C and protein concentration was determined using the bicinchoninic acid (BCA) protein assay kit (PierceTM BCA, Pierce Biotechnology, Rockford, IL, USA).

An equal amount of denatured proteins were separated using 7.5%–15% sodium dodecyl sulfate polyacrylamide gel electrophoresis (SDS-PAGE), and electrophoretically transferred to 0.45 µm polyvinylidene difluoride (PVDF) membrane (Amersham™ Hybond™, GE Healthcare Life Sciences, Freiburg, Germany) as previously described [35]. Membranes were incubated overnight at 4 °C with primary antibodies (listed on Table 2) diluted in 1% milk/ 0.1% PBS-T followed by the incubation with adequate secondary antibodies (Table 2) diluted in 1% milk/ 0.1% PBS-T with agitation for 1 h at room temperature (RT). Finally, membranes were visualized on ImageQuant™LAS500 (GE Healthcare Life Sciences) and optical density of the bands was quantified using Image Quant TL 5.0. software (GE healthcare–Life Sciences, Life Sciences, Freiburg, Germany). Results were normalized against internal controls tubulin or anti-β-actin antibodies and then expressed as percentage of control.

### 2.9. Statistical Analysis

Results were expressed as means ± standard errors of the mean (S.E.M.) and % of the Control, as indicated, using GraphPad Prism^®^ software, version 8.0.1 (GraphPad Software, Inc., La Jolla, CA, USA). Comparisons between groups were analyzed by Student’s *t*-test for normally distributed data or the Mann Whitney test for non-normally distributed data. Differences were considered to be significant at *p* < 0.05.

## 3. Results

### 3.1. Body Weight, Caloric Intake and Glycemic and Insulinemic Profile

**Figure 1:**
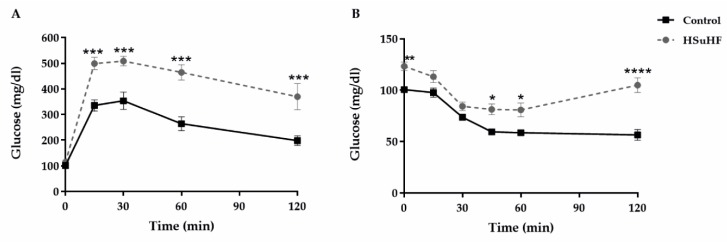
In vivo glucose tolerance test (**A**) and insulin tolerance test (**B**) at the end of the study in the Control and HSuHF-treated rats. Values are means ± S.E.M. of *n* = 8 per group. * *P* < 0.05; ** *P* < 0.01, *** *P* < 0.001 and **** *P* < 0.0001.

Body weight at the beginning of the study was identical between groups. The variation (delta) of BW during the entire treatment was significantly higher (*p* < 0.05) in the HSuHF-treated rats (Table 3). Even though HSuHF-treated animals ingested significantly more liquid and less solid food, a higher total caloric intake was achieved (*p* < 0.001 for all), which was mainly obtained from carbohydrates and lipids. In opposition, the caloric intake obtained from proteins was lower in the prediabetic animals (Table 3). Despite no significant changes between groups on fasting glucose and insulin levels, there were increased values in the HSuHF-treated animals in fed conditions (*p* < 0.001 and *p* < 0.05, respectively), accompanied by impaired GTT and ITT (Figure 1), as viewed by the significantly increased AUCs (Table 3).

### 3.2. Renal Function Data

There was a trend toward reduced kidney weight (KW)/BW ratio in the prediabetic animals versus the control (Table 4). There were no differences between groups regarding serum and urine measures of creatinine and uric acid, as well as concerning their clearances. However, serum and urine BUN levels were significantly decreased (*p* < 0.001) in the HSuHF-treated rats versus the control ones, as well as clearance (*p* < 0.01) (Table 4).

### 3.3. Kidney Histomorphology

**Figure 2:**
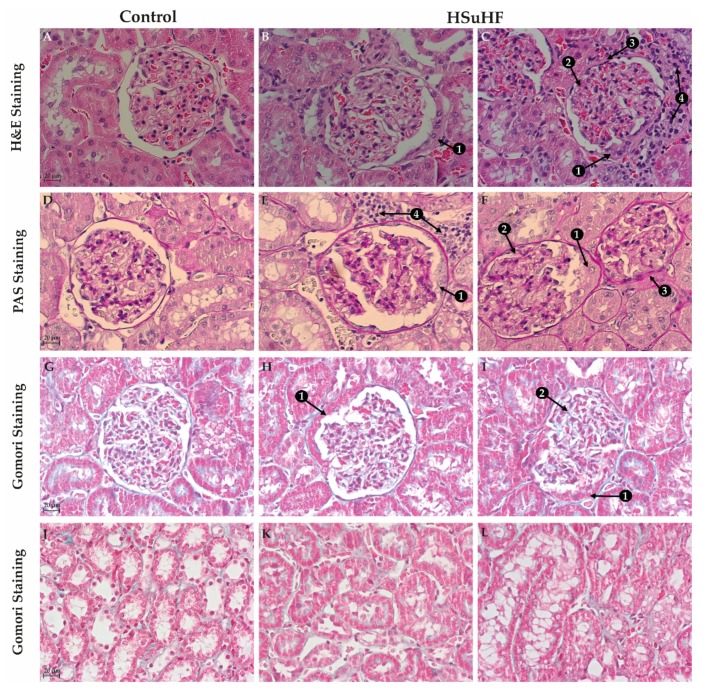
Representative images of glomerular (**A**–**I**) and tubulointerstitial (**J**–**L**) regions and lesions in the Control and HSuHF-treated rats (*n* = 8 per group) evaluate by H&E, PAS and Gomori staining (all with 40× amplification). Arrows represent the glomerular lesions better identified in the above description of results: 1) the presence of glomerular crescent-like structures; 2) mesangial expansion; 3) thickening of the GBM; 4) focal inflammatory infiltrate.

To evaluate the presence (and nature) of glomerular and tubulointerstitial lesions, kidney sections were stained with H&E, PAS, and Gomori staining (Figure 2 and Figure S1). The general observation using H&E staining with 10x magnification suggested the existence of glomerular lesions in the HSuHF animals, without changes of tubulointerstitial structures, when compared with the control kidneys (data not shown), despite the presence of focal inflammatory infiltrate in 2 out of 8 of the HSuHF-fed animals (Figure 2C), located in the tubular interstitial compartment of renal medulla, without signs of interstitial fibrosis and tubular atrophy (IFTA) (Figure 2J–L). The glomerular lesions were further confirmed by using 40x magnification, including thickening of the GBM and mesangial expansion (Figure 2B,C), without glomerular atrophy or glomerulosclerosis, except in one animal of the HSuHF group, most probably more prone to hypercaloric diet (data not shown because were not the most representative lesions). Surprisingly, we found an atypical pattern of extracapillary hyperplasia of glomerular capsule parietal layer surrounding and compressing Bowman’s space in about 20% of the glomeruli of all prediabetic animals, and no more than one per glomerulus (Figure 2B,C and Supplementary Figure S1). These crescent-like structures were even more clearly represented through PAS staining (Figure 2E,F and Supplementary Figure S1), further confirming the existence of focal inflammatory infiltrate, thickening of the GBM and mesangial expansion (Figure 2E,F) in the HSuHF-fed animals.

**Figure 3:**
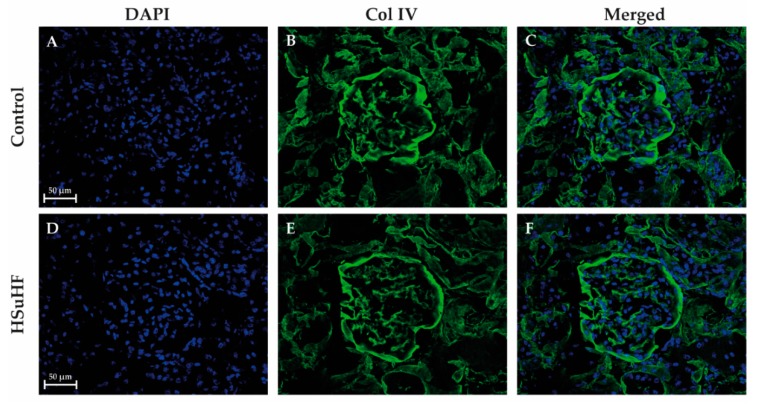
Representative confocal images of immunolabelling for collagen IV (green: **B** and **E**), DAPI (blue: **A** and **D**), as well as merge (**C** and **F**) in the Control and HSuHF-treated rats (*n* = 8 per group).

Gomori staining was unable to show clear evidence of fibrosis in both the Control and HSuHF-fed rats, neither in glomeruli (Figure 2G–I) nor in tubulointerstitial region (Figure 2J–L). This was further confirmed with collagen type IV staining (Figure 3). This type of collagen, particularly the conformations α1, α2, and α6, are normally present in Bowman’s capsule. As expected, Bowman’s capsule was markedly stained as well as the tubular basement membrane and the mesangial matrix [36]. Nonetheless there were no changes between the two groups regarding to the intensity of staining and the integrity of internal glomerular structures. Furthermore, no obvious signs of Bowman’s capsule disruption in renal sections from HSuHF, compared to the control animals, were observed (Figure 3).

**Figure 4:**
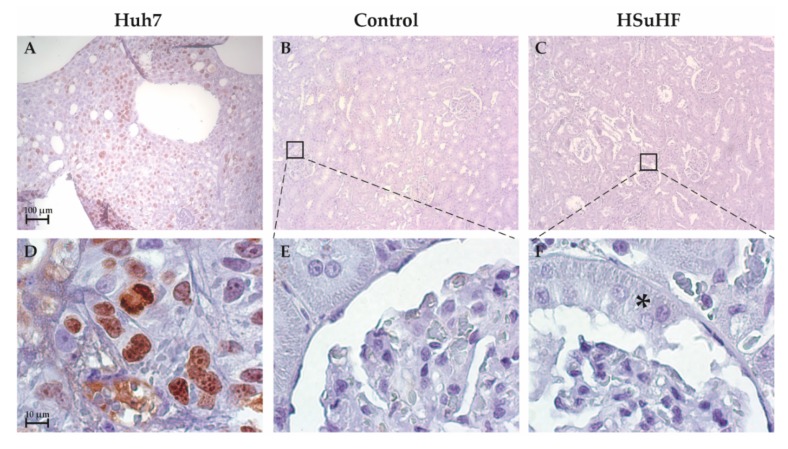
Representative images of Ki-67 immunostaining in the Control (**B** and **E**) and HSuHF-treated (**C** and **F**) rats (*n* = 8 per group). Huh7 cell line was used as a positive control (**A** and **D**). **A**–**C**: 10×; **D**–**F**: 100×). * Crescent-like structure in the HSuHF rats.

To provide an in-depth analysis of crescent-like structures, kidney sections were stained with Ki-67, a classical marker of proliferation (Figure 4). Considering that Ki-67 is crucial for progression of cell division cycle, the absent of staining observed in both glomerular section of control and HSuHF animals (Figure 4) appears to indicate that the renal cells are in the resting state G0. Furthermore, the use of a positive Control (HuH-7 cells, a hepatocellular carcinoma cell line) was essential to corroborate the presence of Ki-67 during mitotic phases.

### 3.4. Serum and Renal Lipids

**Figure 5:**
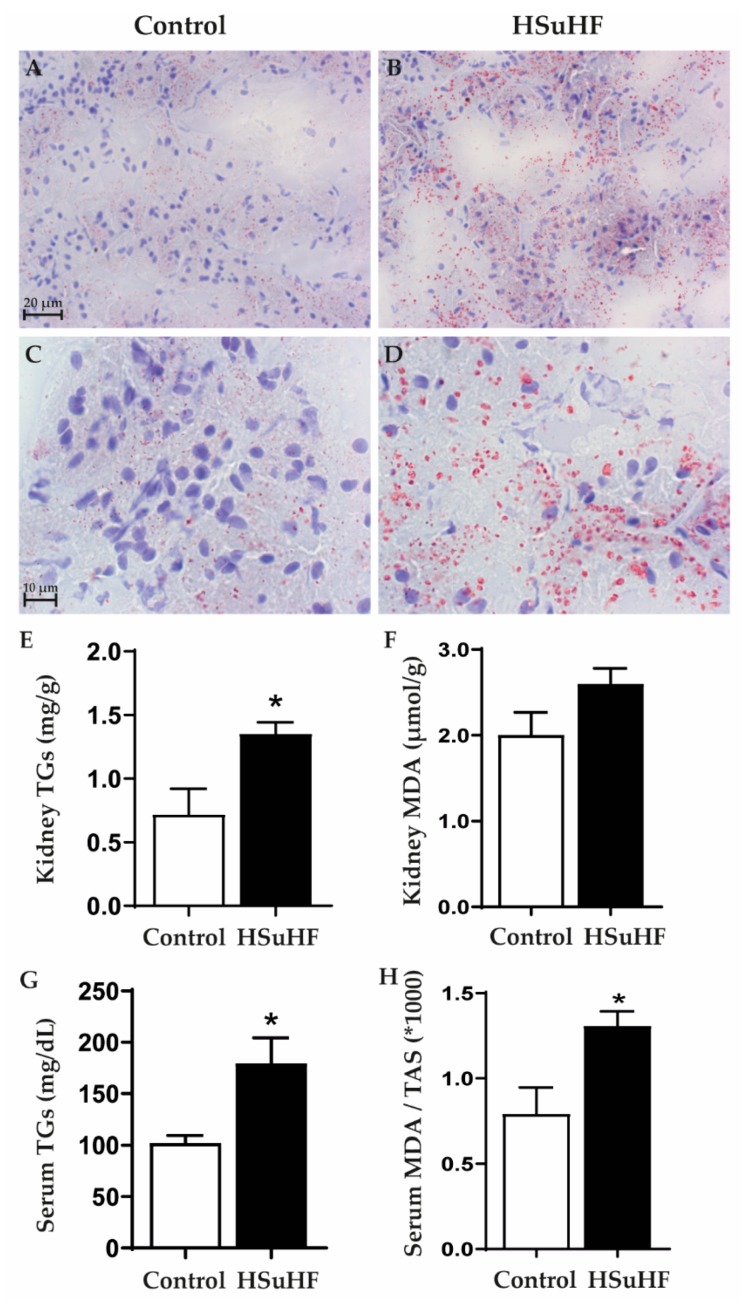
Renal lipid deposition evaluated by Oil-Red O staining in the Control (**A**,**B**) and HSuHF-treated (**C**,**D**) rats; kidney TGs concentration (**E**) and MDA content (**F**); serum TGs concentration (**G**) and MDA/TAS ratio (**H**). Values are means ± S.E.M. of *n* = 8 per group. * *P* < 0.05. **A** and **C** are 40× amplification and **B** and **D** 100×.

There was an increased lipid deposition in the renal tissue of the prediabetic rats, versus the control ones, as viewed by the increased staining with Oil-Red O (Figure 5A–D). The increased lipid deposition was viewed in all the 8 animals of the HSuHF group, and was evident in both renal cortex and medulla. This effect was coherently accompanied by a significantly (*p* < 0.05) increased contents of TGs, both in renal tissue and serum (Figure 5G). In addition, a trend to higher kidney MDA content and a significantly (*p* < 0.05) increased serum MDA/TAS ratio was observed in the HSuHF-treated animals (Figure 5H).

### 3.5. Serum and Renal Inflammatory Markers

**Figure 6:**
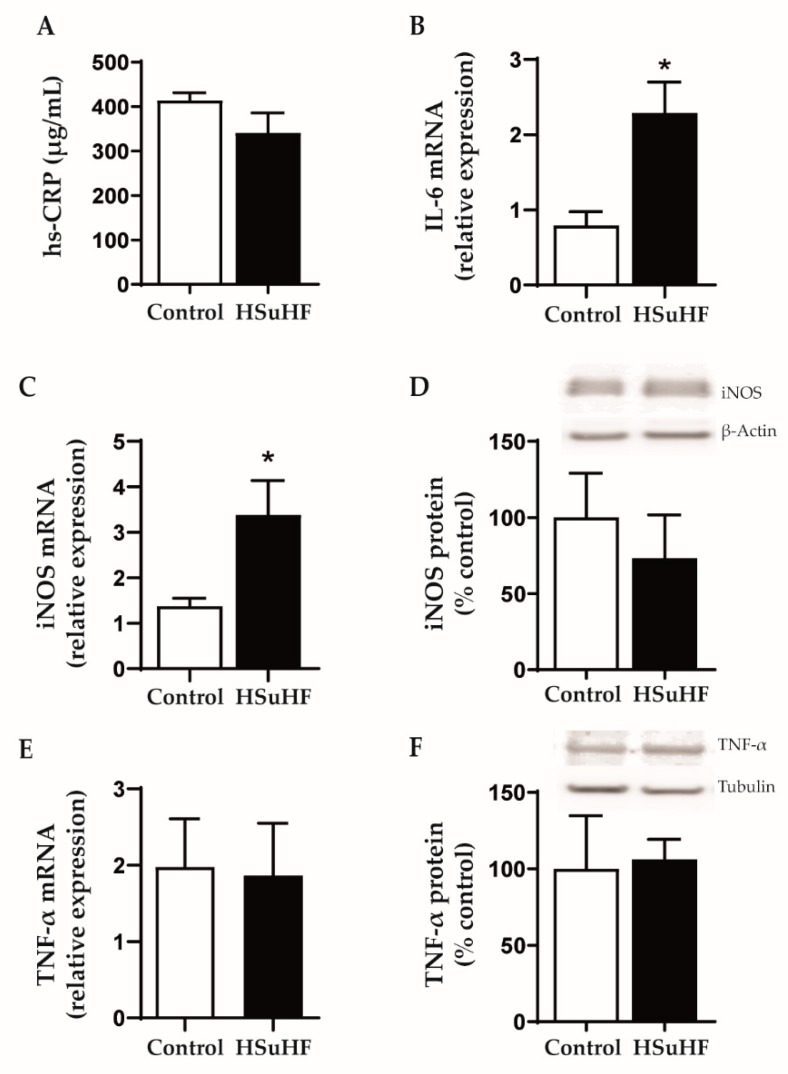
Serum hs-CRP levels (**A**) and kidney expression of IL-6 mRN (**B**), iNOS mRNA (**C**) and protein (**D**), as well as TNF-α mRNA (**E**) and protein (**F**) expression in the Control and HSuHF-treated rats. Values are means ± S.E.M. of *n* = 8 per group (except for western blotting: *n* = 6 per group), and were expressed as relative expression (for mRNA) in relation to two housekeeping genes used (GAPDH and HPRT) and as % of control (for protein). * *P* < 0.05.

There were no changes between groups for serum hs-CRP levels (Figure 6A). However, there was an increased renal expression of IL-6 and iNOS mRNA in the HSuHF-treated animals versus the control ones (Figure 6B,C), without changes of iNOS protein expression (Figure 6D) and TNF-α mRNA and protein expression (Figure 6E,F).

## 4. Discussion

T2DM is the most common cause of chronic kidney disease (CKD) and end stage renal disease (ESRD) worldwide [37]. DN, a long-term major microvascular complication of T1DM and T2DM, affects a large population in the United States and Western Europe, where around one-third of diabetic individuals have nephropathy [38].

Currently existing therapeutic approaches do not allow reversing or controlling the kidney disease in intermediate stages, being clearly ineffective, and trace patients in advanced stages for transplantation or dialysis. This inability to cope with the disease imposes high socio-economic costs that clearly require more effective and, above all, earlier measures. However, for this to become a reality, two conditions are crucial: -on the one hand, finding early markers, more reliable than those currently existing; -on the other, discovering molecular targets for possible therapeutic approaches, namely for the development of new drugs targeting the mechanisms of disease at the kidney level and not only the associated conditions, such as hyperglycemia and hyperlipidemia [6,7]. The scientific knowledge required often demands studies on animal models. It is well recognized, namely by studies in humans, that changes in renal function might start many years before the diagnosis of diabetes, in a prediabetic stage [9,10]. However, much remains to be known regarding the precise phenotype of DN in the prediabetes phase, with a clear lack of information on animal models. In this work, we used a prediabetes rat model induced by a high calorie diet to characterize the phenotype of this early nephropathy.

Male Wistar rats under a hypercaloric diet for 23 weeks showed metabolic features compatible with obese prediabetes. In fact, despite fasting normoglycemia, the HFD-treated rats presented fed hyperglycemia and hyperinsulinemia, with glucose intolerance and reduced insulin sensitivity. Additionally, the animals showed hypertriglyceridemia and increased serum oxidative stress, without changes in hs-CRP. This profile is in line with other studies performed in rats under HFD [22,39].

Concerning renal function, the prediabetic animals showed unchanged values of serum, urine and clearance of creatinine and uric acid, despite reduced BUN values which could be explained by the reduced protein consumption when compared with the control animals. These results are in agreement with other studies in Wistar rats under longstanding HFD that were unable to show marked changes on renal function markers [40]. The absence of changes in creatinine until a significant part of the glomeruli is already affected (it is estimated about 50%) is a known fact that has been portrayed as a limitation of the classic markers of renal function assessment, strongly recommending better markers of early renal dysfunction [12,13].

Regarding the histomorphological characterization, no significant tubulointerstitial lesions were observed, despite the presence of focal inflammatory infiltrate in 2 out of 8 of the HsuHF-fed animals, located in the tubular interstitial compartment of renal medulla, without signs of IFTA. However, we found the existence of several glomerular lesions, including mesangial expansion and thickening of the GBM, without atrophy or glomerulosclerosis (except in one animal), which collectively is in agreement with other models in initial stages of nephropathy [22]. Remarkably, we found an atypical pattern of extracapillary hyperplasia of glomerular capsule parietal layer surrounding and compressing Bowman’s space in about 20% of the glomeruli of all prediabetic animals. These lesions, resembling crescent-like structures, are a well-known pathological component of glomerulonephritis, affecting nearly 50% of overall glomerular circumference [41,42]. Yet, glomerular crescents development within diabetic kidney disease (DKD) has also been reported and is envisaged as a regular feature of glomerular changes within diabetes, although the available information is largely limited to the examination of human biopsies [43,44,45,46,47]. It is important to highlight that renal biopsies in diabetes are reserved for patients with atypical presentations in routinely clinical practice. Thus, qualitative and quantitative renal morphology information of human diabetic glomerular injuries greatly involve an intrinsic selection bias, reinforcing the importance of preclinical assessment of DKD through reliable animal models [45,48,49]. Interestingly, a linkage between diabetic glomerulopathy and crescent-like structures has already been pinpointed in Zucker diabetic fatty (ZDF) rats, a genetic T2DM animal model widely used in the preclinical setting as they spontaneously develop insulin resistance and obesity at a young age and progressively develop hyperglycemia and renal injury [20,50,51]. Remarkably, extracapillary hyperplasia within glomerular capsule was observed in nearly 30% of glomeruli of young ZDF rats (7 weeks old) displaying glucose intolerance (although still normoglycemic) and impaired renal function (increased urinary protein/creatinine ratio). An aggravation of the metabolic status until overt diabetes (week 20) was accompanied by a progressive deterioration of renal function along with glomerular histopathological features comprising increased crescent-like structures in Bowman’s parietal layer (≈44%) [52]. Likewise, we here report glomerular crescent-like structures paralleling impaired renal function in a diet-induced animal model of prediabetes without overt hyperglycemia. Crescents have been classically defined as any proliferative or fibrous space within parietal layer of Bowman’s space [47]. Even though the precise pathogenesis of crescents‘ formation is unknown, two types are generally recognized: inflammatory crescents and pseudocrescents. An elegant work from Gaut et al. (2014) provided valuable evidence of the nature of cells forming crescents in diabetes, composed mainly by parietal epithelial cells (PEC’s) and podocytes within the parietal layer of glomerular capsule without GBM rupture, an ultrastructural alteration that is typically observed in inflammatory crescents [53]. These authors postulate that within DKD, pseudocrescents are the most likely scenario. In an attempt to evaluate the nature of these lesions in our experimental setting, we started to assess fibrotic features through the classical Gomori’s Green Trichrome staining. We were unable to visualize evident signs of extracellular matrix accumulation, mainly composed by proteoglycans, fibronectin and fibrillar collagen [54]. Since trichrome stains is often unreliable at milder levels of fibrosis [55], we further analyze conformational determinants on collagen type IV, normally used to demarcate Bowman’s capsule in renal tissues [56]. Consistent with the lack of glomerular sclerotic lesions in our previous histological analysis, there were no evident signs of collagen IV structural reorganization. We proceed with the identification of proliferating cellular subpopulations through Ki-67 immunohistochemistry. Cells express Ki-67 antigen during S, G2 and M phases while it is undetectable at quiescent stages [57,58]. The lack of Ki-67 staining hints for a post-proliferating quiescent state of cellular crescentic components at the time point of our study. Future studies, namely in earlier time-points and using alternative markers of proliferation (e.g., BrdU), are warranted to unequivocally conclude the proliferating nature of crescents as well as their cellular composition.

The varied terminology used to describe these structures (crescents, pseudocrescents, extracapillary hypercellularity) clearly shows that their origin, biology, and pathophysiological implications remain doubtful and should be further elucidated [46,47]. Among the various hypotheses that have been suggested to explain the pathophysiological role of pseudocrescents, the one that seems to be more relevant is that these structures may represent a potentially beneficial response to the insult. Briefly, it has been described that PECs may have the capacity to act as progenitor cells for injured podocytes and migrate from Bowman’s capsule to the capillary tuft in response to injury. This ability has been envisaged as an attempt of the injured glomerulus to heal itself and counteract the ongoing injury through podocytes repopulation [46,53]. In our model, hyperglycemia or hyperlipidemia induced by HSuHF diet might be the driven forces for the metabolic damage (via glucotoxicity and lipotoxicity) underlying the formation of psudocrescents. Despite the absence of a clear inflammatory phenotype, at both systemic and renal levels, the existence of oxidative stress and renal lipidosis might explain the renal damage, as previously suggested by others [59,60]. These possibilities are in line with the association between obesity/adiposity and increased risk of GFR decline and mortality in individuals with and without CKD [27]. Renal damage induced by lipotoxicity is a complex process that has been progressively disclosed [60,61]. The increased renal expression of IL-6, regardless of normal serum hs-CRP, might be linked to hyperglycemic and hyperlipidemic stimuli, as previously suggested [62,63]. It has becoming clear that changes in renal lipid metabolism promote ectopic lipid deposition in the kidney tissue, which contributes to nephropathy. In fact, para-perirenal distribution of fat was associated with reduced GFR, which might occur via distinct mechanisms, including by adverse effects caused by increased leptin availability or free-fatty acids migration into the kidney, leading to renal lipidosis, as well as by direct mechanical compression of renal vessels and parenchyma with subsequent reduced renal blood flow [59]. Further studies should focus on the elucidation of the precise role of lipid deposition within the renal tissue for the evolution of renal damage and the early appearance of pseudocrescents. In any case, PEC’s hyperplasia co-expressing kidney injury molecule-1 expression have been associated with progressive podocytopenia in diabetic rats [52]. Moreover, crescent-like lesions have been identified as a novel poor prognostic indicator of time from DKD to ESRD [46].

## 5. Conclusions and Future Directions

We here presented a new rat model of hypercaloric diet-induced prediabetes with renal damage. Such a model may provide a valuable tool to evaluate early features of DN and disclose new predictors of disease progression, with focus on crescent-like lesions, which are an early signature that deserves in-depth elucidation regarding their origin, biology, and pathophysiological implications. Overall, it will be paramount in future studies to early identify them and elucidate the molecular mechanisms that govern their appearance and growth, in order to find approaches that enhance their putative endogenous repair ability before a condition of exhaustion occurs, after which nephropathy might progress to advanced chronic stages. Specifically, it will be important to clarify the cell composition of crescents, namely using molecular markers for different cell types, including PECs (e.g., PAX-2, PAX-8, cytokeratin), podocytes (e.g., synaptopodin, VEGF, CD36) and/or GBM (e.g., claudin-1) [64]. Furthermore, the impact of lipid accumulation in renal structures for the development of renal disease, namely as inductor of inflammatory markers (such as IL-6), should be further elucidated. The precise determination of lipids (including non-esterified fatty acids) accumulated in glomeruli and tubules will be potentially relevant to better characterize the renal disease at this stage of prediabetes.

Despite the possible disadvantages of this model of nephropathy in a condition of prediabetes, namely the long-lasting (23 weeks) feeding with a hypercaloric diet, which is associated with a significant expenditure of human and financial resources when compared with genetic models with fast development of DN, significant advantages exist. In fact, the hypercaloric diet may be suspended at any time throughout treatment, thus allowing testing the possibility of renal disease reversal, which open up the possibility to study therapeutic strategies targeting kidney recovering. However, further studies are required to evaluate intermediate time-points and better characterize the evolution of kidney dysfunction and lesions during the progression of prediabetes. Additional topics for future work include the evaluation of female rats in order to confirm or deny sex susceptibility to this phenotype, as has been suggested in other models.

## Figures and Tables

**Table 1:** Primer sequence used for the RT-PCR analysis.

Gene	Forward Primer Sequence	Reverse	T (°C)
iNOS	AGAGACAGAAGTGCGATC	AGATTCAGTAGTCCACAATAGTA	60
IL-6	GGAGAAGTTAGAGTCACAGA	GCCGAGTAGACCTCATAG	60
TNF-α	CCCAATCTGTGTCCTTCT	TTCTGAGCATCGTAGTTGT	60
GAPDH	GACTTCAACAGCAACTCC	GCCATATTCATTGTCATACCA	60
HPRT	ATGGGAGGCCATCACATTGT	ATGTAATCCAGCAGGTCAGCAA	58

**Table 2:** Primary and secondary antibodies used for Western Blotting analysis.

Antibody	Dilution	Catalog No.-Company
iNOS	1:1000	Ab178945-Abcam
TNF-α	1:500	Ab66579-Abcam
β-actin	1:1000	A5316-Sigma-Aldrich
Tubulin	1:1000	AB0046-200-SICGEN
Goat anti-mouse IgG-HRP	1:10,000	R-05071-Advansta
Goat anti-rabbit IgG-HRP	1:10,000	R-05072-Advansta

**Table 3:** Body weight, caloric intake and glycemic and insulinemic profile.

Parameters	Control	HSuHF
**Body Weight (BW)**		
Initial (g)	300.00 ± 10.88	297.11 ± 9.71
Final (g)	507.57 ± 18.15	578.62 ± 32.89
Delta BW (g)	228.50 ± 10.04	311.20 ± 21.11*
**Caloric Data**		
Food Intake (g/week)	163.60 ± 2.20	77.11 ± 0.54 ***
Beverage Intake (mL/week)	211.80 ± 3.69	326.70 ± 50.89 ***
Calories from Carbohydrates (kcal/rat/week)	350.40 ± 3.89	574.50 ± 13.84 ***
Calories from Lipids (kcal/rat/week)	44.22 ± 0.47	97.74 ± 8.71
Calories from Proteins (kcal/rat/week)	121.20 ± 1.29	51.68 ± 1.60 ***
∑ Total calories (kcal/rat/week)	515.80 ± 5.49	713.40 ± 10.17 ***
**Glycemic Profile**		
Fasting glucose(mg/dL)	100.50 ± 1.37	115.77 ± 1.42
Fed glucose (mg/dL)	149.14 ± 7.95	273.14 ± 33.53 ***
GTT (AUC)	31856 ± 2565	54974.2 ± 3413 ***
HbA1c (%)	3.73 ± 0.05	4.00 ± 0.15
**Insulinemic Profile**		
Fasting insulin (µg/L)	0.577 ± 0.082	1.454 ± 0.197
Fed insulin (µg/L)	0.899 ± 0.159	2.426 ± 0.535 *
ITT (AUC)	8107 ± 299	11175 ± 587 **

Values are means ± S.E.M. of *n* = 8 per group. * *P* < 0.05; ** *P* < 0.01 and *** *P* < 0.001.

**Table 4:** Kidney weight and serum and urine measures of renal function.

Parameters	Control	HSuHF
**Kidney Weight (KW)**		
Absolute (g)	2.86 ± 0.13	2.90 ± 0.12
KW/BW (g/kg)	5.63 ± 0.22	4.92 ± 0.07
**Creatinine**		
Serum (mg/dL)	0.36 ± 0.01	0.38 ± 0.02
Urine (mg/dL)	100.40 ± 14.78	61.15 ± 7.84
Clearance (mL/h)	200.40 ± 18.55	184.4 ± 11.57
**BUN**		
Serum (mg/dL)	14.19 ± 0.58	8.57 ± 0.60 ***
Urine (mg/dL)	1554.00 ± 132.80	493.00 ± 82.90 ***
Clearance (mL/h)	92.53 ± 7.24	35.59 ± 4.29 **
**Uric Acid**		
Serum (mg/dL)	1.79 ± 0.33	2.33 ± 0.52
Urine (mg/dL)	12.80 ± 1.74	9.86 ± 0.69
Clearance (mL/h)	6.50 ± 1.10	6.00 ± 1.02

Values are means ± S.E.M. of *n* = 8 per group. ** *P* < 0.01 and *** *P* < 0.001.

## Supplementary Materials

The following are available online at https://www.mdpi.com/2072-6643/12/4/881/s1, Figure S1: Images of glomerular crescent-like structures (depicted with arrows) evaluated by H&E, PAS and Gomori staining in the HSuHF-treated rats.

## References

[1] Federation I.D. (2019). IDF Diabetes Atlas.

[2] Reutens A.T., Atkins R.C. (2011). Epidemiology of diabetic nephropathy. Contrib. Nephrol..

[3] Spijkerman A.M., Dekker J.M., Nijpels G., Adriaanse M.C., Kostense P.J., Ruwaard D., Stehouwer C.D., Bouter L.M., Heine R.J. (2003). Microvascular complications at time of diagnosis of type 2 diabetes are similar among diabetic patients detected by targeted screening and patients newly diagnosed in general practice: The hoorn screening study. Diabetes Care.

[4] Stehouwer C.D.A. (2018). Microvascular Dysfunction and Hyperglycemia: A Vicious Cycle With Widespread Consequences. Diabetes.

[5] Arora M.K., Singh U.K. (2013). Molecular mechanisms in the pathogenesis of diabetic nephropathy: An update. Vasc. Pharmacol..

[6] Valencia W.M., Florez H. (2017). How to prevent the microvascular complications of type 2 diabetes beyond glucose control. BMJ.

[7] Barrett E.J., Liu Z., Khamaisi M., King G.L., Klein R., Klein B.E.K., Hughes T.M., Craft S., Freedman B.I., Bowden D.W. (2017). Diabetic Microvascular Disease: An Endocrine Society Scientific Statement. J. Clin. Endocrinol. Metab..

[8] Tabak A.G., Herder C., Rathmann W., Brunner E.J., Kivimaki M. (2012). Prediabetes: A high-risk state for diabetes development. Lancet.

[9] Melsom T., Schei J., Stefansson V.T., Solbu M.D., Jenssen T.G., Mathisen U.D., Wilsgaard T., Eriksen B.O. (2016). Prediabetes and Risk of Glomerular Hyperfiltration and Albuminuria in the General Nondiabetic Population: A Prospective Cohort Study. Am. J. Kidney Dis..

[10] Markus M.R.P., Ittermann T., Baumeister S.E., Huth C., Thorand B., Herder C., Roden M., Siewert-Markus U., Rathmann W., Koenig W. (2018). Prediabetes is associated with microalbuminuria, reduced kidney function and chronic kidney disease in the general population: The KORA (Cooperative Health Research in the Augsburg Region) F4-Study. Nutr. Metab. Cardiovasc. Dis..

[11] Echouffo-Tcheugui J.B., Narayan K.M., Weisman D., Golden S.H., Jaar B.G. (2016). Association between prediabetes and risk of chronic kidney disease: A systematic review and meta-analysis. Diabet. Med..

[12] Ferguson M.A., Waikar S.S. (2012). Established and emerging markers of kidney function. Clin. Chem..

[13] Waikar S.S., Betensky R.A., Bonventre J.V. (2009). Creatinine as the gold standard for kidney injury biomarker studies?. Nephrol. Dial. Transpl..

[14] Currie G., McKay G., Delles C. (2014). Biomarkers in diabetic nephropathy: Present and future. World J. Diabetes.

[15] Soler M.J., Riera M., Batlle D. (2012). New experimental models of diabetic nephropathy in mice models of type 2 diabetes: Efforts to replicate human nephropathy. Exp. Diabetes Res..

[16] Kaur M., Bedi O., Sachdeva S., Reddy B.V., Kumar P. (2014). Rodent animal models: From mild to advanced stages of diabetic nephropathy. Inflammopharmacology.

[17] Betz B., Conway B.R. (2016). An Update on the Use of Animal Models in Diabetic Nephropathy Research. Curr. Diabetes Rep..

[18] Tervaert T.W., Mooyaart A.L., Amann K., Cohen A.H., Cook H.T., Drachenberg C.B., Ferrario F., Fogo A.B., Haas M., de Heer E. (2010). Pathologic classification of diabetic nephropathy. J. Am. Soc. Nephrol..

[19] Brosius F.C., Alpers C.E., Bottinger E.P., Breyer M.D., Coffman T.M., Gurley S.B., Harris R.C., Kakoki M., Kretzler M., Leiter E.H. (2009). Mouse models of diabetic nephropathy. J. Am. Soc. Nephrol..

[20] Kong L.L., Wu H., Cui W.P., Zhou W.H., Luo P., Sun J., Yuan H., Miao L.N. (2013). Advances in murine models of diabetic nephropathy. J. Diabetes Res..

[21] Kitada M., Ogura Y., Koya D. (2016). Rodent models of diabetic nephropathy: Their utility and limitations. Int. J. Nephrol. Renovasc. Dis..

[22] Preguica I., Alves A., Nunes S., Gomes P., Fernandes R., Viana S.D., Reis F. (2020). Diet-Induced Rodent Models of Diabetic Peripheral Neuropathy, Retinopathy and Nephropathy. Nutrients.

[23] Burgeiro A., Cerqueira M.G., Varela-Rodriguez B.M., Nunes S., Neto P., Pereira F.C., Reis F., Carvalho E. (2017). Glucose and Lipid Dysmetabolism in a Rat Model of Prediabetes Induced by a High-Sucrose Diet. Nutrients.

[24] Nunes S., Soares E., Fernandes J., Viana S., Carvalho E., Pereira F.C., Reis F. (2013). Early cardiac changes in a rat model of prediabetes: Brain natriuretic peptide overexpression seems to be the best marker. Cardiovasc. Diabetol..

[25] Martin-Cordero L., Galvez I., Hinchado M.D., Ortega E. (2019). beta2 Adrenergic Regulation of the Phagocytic and Microbicide Capacity of Macrophages from Obese and Lean Mice: Effects of Exercise. Nutrients.

[26] Galvez I., Martin-Cordero L., Hinchado M.D., Alvarez-Barrientos A., Ortega E. (2019). Obesity Affects beta2 Adrenergic Regulation of the Inflammatory Profile and Phenotype of Circulating Monocytes from Exercised Animals. Nutrients.

[27] Chang A.R., Grams M.E., Ballew S.H., Bilo H., Correa A., Evans M., Gutierrez O.M., Hosseinpanah F., Iseki K., Kenealy T. (2019). Adiposity and risk of decline in glomerular filtration rate: Meta-analysis of individual participant data in a global consortium. BMJ.

[28] Whaley-Connell A., Sowers J.R. (2017). Obesity and kidney disease: From population to basic science and the search for new therapeutic targets. Kidney Int..

[29] Tziomalos K., Athyros V.G. (2015). Diabetic Nephropathy: New Risk Factors and Improvements in Diagnosis. Rev. Diabet. Stud..

[30] Gutwein P., Abdel-Bakky M.S., Doberstein K., Schramme A., Beckmann J., Schaefer L., Amann K., Doller A., Kampfer-Kolb N., Abdel-Aziz A.A. (2009). CXCL16 and oxLDL are induced in the onset of diabetic nephropathy. J. Cell Mol. Med..

[31] Wang Y.C., Feng Y., Lu C.Q., Ju S. (2018). Renal fat fraction and diffusion tensor imaging in patients with early-stage diabetic nephropathy. Eur. Radiol..

[32] Moreno J.A., Gomez-Guerrero C., Mas S., Sanz A.B., Lorenzo O., Ruiz-Ortega M., Opazo L., Mezzano S., Egido J. (2018). Targeting inflammation in diabetic nephropathy: A tale of hope. Expert Opin. Investig. Drugs.

[33] Ferreira L., Teixeira-de-Lemos E., Pinto F., Parada B., Mega C., Vala H., Pinto R., Garrido P., Sereno J., Fernandes R. (2010). Effects of sitagliptin treatment on dysmetabolism, inflammation, and oxidative stress in an animal model of type 2 diabetes (ZDF rat). Mediators Inflamm..

[34] Pestel S., Krzykalla V., Weckesser G. (2007). Measurement of glomerular filtration rate in the conscious rat. J. Pharmacol. Toxicol. Methods.

[35] Viana S.D., Fernandes R.C., Canas P.M., Silva A.M., Carvalho F., Ali S.F., Fontes Ribeiro C.A., Pereira F.C. (2016). Presymptomatic MPTP Mice Show Neurotrophic S100B/mRAGE Striatal Levels. CNS Neurosci. Ther..

[36] Liu Y.J., Lin Y.C., Lee J.C., Kuo S.C., Ho C.T., Huang L.J., Kuo D.H., Way T.D. (2014). CCT327 enhances TRAIL-induced apoptosis through the induction of death receptors and downregulation of cell survival proteins in TRAIL-resistant human leukemia cells. Oncol. Rep..

[37] Glassock R.J., Warnock D.G., Delanaye P. (2017). The global burden of chronic kidney disease: Estimates, variability and pitfalls. Nat. Rev. Nephrol..

[38] Jha V., Garcia-Garcia G., Iseki K., Li Z., Naicker S., Plattner B., Saran R., Wang A.Y., Yang C.W. (2013). Chronic kidney disease: Global dimension and perspectives. Lancet.

[39] Marques C., Meireles M., Norberto S., Leite J., Freitas J., Pestana D., Faria A., Calhau C. (2016). High-fat diet-induced obesity Rat model: A comparison between Wistar and Sprague-Dawley Rat. Adipocyte.

[40] Rangel Silvares R., Nunes Goulart da Silva Pereira E., Eduardo Ilaquita Flores E., Lino Rodrigues K., Ribeiro Silva A., Goncalves-de-Albuquerque C.F., Daliry A. (2019). High-fat diet-induced kidney alterations in rats with metabolic syndrome: Endothelial dysfunction and decreased antioxidant defense. Diabetes Metab. Syndr. Obes..

[41] Jennette J.C. (2003). Rapidly progressive crescentic glomerulonephritis. Kidney Int..

[42] Haas M., Verhave J.C., Liu Z.H., Alpers C.E., Barratt J., Becker J.U., Cattran D., Cook H.T., Coppo R., Feehally J. (2017). A Multicenter Study of the Predictive Value of Crescents in IgA Nephropathy. J. Am. Soc. Nephrol..

[43] Wakabayashi N., Takeda S., Imai T., Akimoto T., Nagata D. (2015). Unexpected observation of glomerular crescents in a patient with diabetes who developed drug-induced acute tubulointerstitial nephritis: A possible feature of diabetic nephropathy?. Nephrology.

[44] Toth T. (1987). Epithelial crescent in diabetic glomeruli. A case report. Int. Urol. Nephrol..

[45] Otani N., Akimoto T., Yumura W., Matsubara D., Iwazu Y., Numata A., Miki T., Takemoto F., Fukushima N., Muto S. (2012). Is there a link between diabetic glomerular injury and crescent formation? A case report and literature review. Diagn. Pathol..

[46] Mottl A.K., Gasim A., Schober F.P., Hu Y., Dunnon A.K., Hogan S.L., Jennette J.C. (2018). Segmental Sclerosis and Extracapillary Hypercellularity Predict Diabetic ESRD. J. Am. Soc. Nephrol..

[47] Elfenbein I.B., Reyes J.W. (1975). Crescents in diabetic glomerulopathy. Incidence and clinical significance. Lab. Investig..

[48] Nasr S.H., D’Agati V.D., Said S.M., Stokes M.B., Appel G.B., Valeri A.M., Markowitz G.S. (2008). Pauci-immune crescentic glomerulonephritis superimposed on diabetic glomerulosclerosis. Clin. J. Am. Soc. Nephrol..

[49] Mazzucco G., Bertani T., Fortunato M., Bernardi M., Leutner M., Boldorini R., Monga G. (2002). Different patterns of renal damage in type 2 diabetes mellitus: A multicentric study on 393 biopsies. Am. J. Kidney Dis..

[50] Marques C., Mega C., Goncalves A., Rodrigues-Santos P., Teixeira-Lemos E., Teixeira F., Fontes-Ribeiro C., Reis F., Fernandes R. (2014). Sitagliptin prevents inflammation and apoptotic cell death in the kidney of type 2 diabetic animals. Mediators Inflamm..

[51] Mega C., de Lemos E.T., Vala H., Fernandes R., Oliveira J., Mascarenhas-Melo F., Teixeira F., Reis F. (2011). Diabetic nephropathy amelioration by a low-dose sitagliptin in an animal model of type 2 diabetes (Zucker diabetic fatty rat). Exp. Diabetes Res..

[52] Zhao X., Zhang Y., Li L., Mann D., Imig J.D., Emmett N., Gibbons G., Jin L.M. (2011). Glomerular expression of kidney injury molecule-1 and podocytopenia in diabetic glomerulopathy. Am. J. Nephrol..

[53] Gaut J.P., Hoshi M., Jain S., Liapis H. (2014). Claudin 1 and nephrin label cellular crescents in diabetic glomerulosclerosis. Hum. Pathol..

[54] Kaissling B., Le Hir M. (2008). The renal cortical interstitium: Morphological and functional aspects. Histochem. Cell Biol..

[55] Farris A.B., Alpers C.E. (2014). What is the best way to measure renal fibrosis?: A pathologist’s perspective. Kidney Int. Suppl..

[56] Roeder S.S., Stefanska A., Eng D.G., Kaverina N., Sunseri M.W., McNicholas B.A., Rabinovitch P., Engel F.B., Daniel C., Amann K. (2015). Changes in glomerular parietal epithelial cells in mouse kidneys with advanced age. Am. J. Physiol. Renal Physiol..

[57] Miller I., Min M., Yang C., Tian C., Gookin S., Carter D., Spencer S.L. (2018). Ki67 is a Graded Rather than a Binary Marker of Proliferation versus Quiescence. Cell Rep..

[58] Zambon A.C. (2010). Use of the Ki67 promoter to label cell cycle entry in living cells. Cytom. A.

[59] Geraci G., Zammuto M.M., Mattina A., Zanoli L., Geraci C., Granata A., Nardi E., Fatuzzo P.M., Cottone S., Mule G. (2018). Para-perirenal distribution of body fat is associated with reduced glomerular filtration rate regardless of other indices of adiposity in hypertensive patients. J. Clin. Hypertens..

[60] Markova I., Miklankova D., Huttl M., Kacer P., Skibova J., Kucera J., Sedlacek R., Kacerova T., Kazdova L., Malinska H. (2019). The Effect of Lipotoxicity on Renal Dysfunction in a Nonobese Rat Model of Metabolic Syndrome: A Urinary Proteomic Approach. J. Diabetes Res..

[61] Izquierdo-Lahuerta A., Martinez-Garcia C., Medina-Gomez G. (2016). Lipotoxicity as a trigger factor of renal disease. J. Nephrol..

[62] Feigerlova E., Battaglia-Hsu S.F. (2017). IL-6 signaling in diabetic nephropathy: From pathophysiology to therapeutic perspectives. Cytokine Growth Factor Rev..

[63] De Vries A.P., Ruggenenti P., Ruan X.Z., Praga M., Cruzado J.M., Bajema I.M., D’Agati V.D., Lamb H.J., Pongrac Barlovic D., Hojs R. (2014). Fatty kidney: Emerging role of ectopic lipid in obesity-related renal disease. Lancet Diabetes Endocrinol..

[64] Dijkman H.B., Weening J.J., Smeets B., Verrijp K.C., van Kuppevelt T.H., Assmann K.K., Steenbergen E.J., Wetzels J.F. (2006). Proliferating cells in HIV and pamidronate-associated collapsing focal segmental glomerulosclerosis are parietal epithelial cells. Kidney Int..

