# Supplementary material for: Crescent-Like Lesions as an Early Signature of Nephropathy in a Rat Model of Prediabetes Induced by a Hypercaloric Diet
Source: Nutrients. 2020 Mar 25;12(4):881. doi: 10.3390/nu12040881 (PMC7230605; doi:10.3390/nu12040881)
Supplement: Supplementary file 1 [file nutrients-12-00881-s001.pdf]

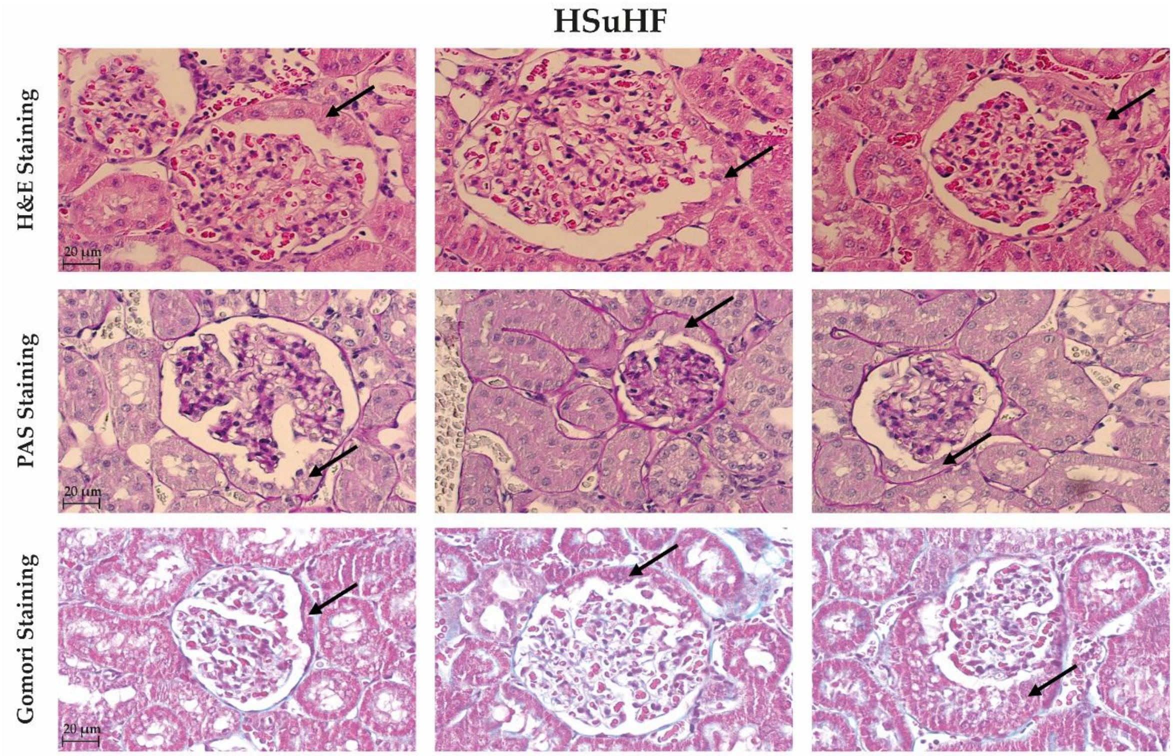

Supplementary Figure 1. Images of glomerular crescent-like structures (depicted with arrows) evaluated by H&E, PAS and Gomori staining in the HSuHF-treated rats.
